# *Komagataella phaffii* as a Platform for Heterologous Expression of Enzymes Used for Industry

**DOI:** 10.3390/microorganisms12020346

**Published:** 2024-02-07

**Authors:** Tamara M. Khlebodarova, Natalia V. Bogacheva, Andrey V. Zadorozhny, Alla V. Bryanskaya, Asya R. Vasilieva, Danil O. Chesnokov, Elena I. Pavlova, Sergey E. Peltek

**Affiliations:** 1 Kurchatov Genomic Center at Institute of Cytology and Genetics, Siberian Branch of Russian Academy of Sciences, 630090 Novosibirsk, Russia; tamara@bionet.nsc.ru (T.M.K.); bogatcheva@bionet.nsc.ru (N.V.B.); evergreen@bionet.nsc.ru (A.V.Z.); alla@bionet.nsc.ru (A.V.B.); vasilieva@bionet.nsc.ru (A.R.V.); 2 Laboratory Molecular Biotechnologies of the Federal Research Center Institute of Cytology and Genetics, Siberian Branch of Russian Academy of Sciences, 630090 Novosibirsk, Russia; 3 Sector of Genetics of Industrial Microorganisms of Federal Research Center Institute of Cytology and Genetics, Siberian Branch of Russian Academy of Sciences, 630090 Novosibirsk, Russia; chesnokovdo@bionet.nsc.ru (D.O.C.); pavlovaey@bionet.nsc.ru (E.I.P.)

**Keywords:** *Komagataella phaffii*, promoters of heterologous proteins, expression, signal sequence, protein production, post-translational modifications

## Abstract

In the 1980s, *Escherichia coli* was the preferred host for heterologous protein expression owing to its capacity for rapid growth in complex media; well-studied genetics; rapid and direct transformation with foreign DNA; and easily scalable fermentation. Despite the relative ease of use of *E. coli* for achieving the high expression of many recombinant proteins, for some proteins, e.g., membrane proteins or proteins of eukaryotic origin, this approach can be rather ineffective. Another microorganism long-used and popular as an expression system is baker’s yeast, *Saccharomyces cerevisiae*. In spite of a number of obvious advantages of these yeasts as host cells, there are some limitations on their use as expression systems, for example, inefficient secretion, misfolding, hyperglycosylation, and aberrant proteolytic processing of proteins. Over the past decade, nontraditional yeast species have been adapted to the role of alternative hosts for the production of recombinant proteins, e.g., *Komagataella phaffii*, *Yarrowia lipolytica*, and *Schizosaccharomyces pombe*. These yeast species’ several physiological characteristics (that are different from those of *S. cerevisiae*), such as faster growth on cheap carbon sources and higher secretion capacity, make them practical alternative hosts for biotechnological purposes. Currently, the *K. phaffii*-based expression system is one of the most popular for the production of heterologous proteins. Along with the low secretion of endogenous proteins, *K. phaffii* efficiently produces and secretes heterologous proteins in high yields, thereby reducing the cost of purifying the latter. This review will discuss practical approaches and technological solutions for the efficient expression of recombinant proteins in *K. phaffii*, mainly based on the example of enzymes used for the feed industry.

## 1. Introduction

In the 1980s, *Escherichia coli* was the preferred host for heterologous protein expression owing to its capacity for rapid growth (cell doubling time: 20 min) in complex media; well-studied genetics, physiology, and metabolism; rapid and direct transformation with foreign DNA; and easily scalable fermentation [1,2,3,4,5]. Despite the relative ease of use of *E. coli* for achieving high expression of many recombinant proteins, for some proteins, e.g., proteins of eukaryotic origin and membrane proteins, this approach can be a rather labor-intensive and ineffective endeavor. In this case, there are difficulties with protein folding and with its secretion as a consequence of the formation of inclusion bodies due to poor protein solubility [6,7]. Furthermore, certain limitations exist in terms of the ability of *E. coli* to ferment under conditions of high cell density [8,9]. One of the main disadvantages of using *E. coli* strains for commercial enzyme production is the safety concerns associated with the lack of the GRAS (generally recognized as safe) status of this bacterium. Nonetheless, so far, interest in *E. coli* has not disappeared from the standpoint of its application to the expression of heterologous proteins (see review: [10]).

Another microorganism long-used and popular as an expression system is baker’s yeast, *Saccharomyces cerevisiae*, which is the best-characterized eukaryotic organism for the production of heterologous proteins [11]. *S. cerevisiae* is an industrial microorganism with the GRAS status [12]. The *S. cerevisiae*-based expression system is similar to that of higher eukaryotes and can perform some post-translational modifications and secrete a target protein [13], thereby making this species a preferred host for the production of recombinant proteins, especially those derived from higher eukaryotes. The ability of yeast to secrete protein products has many advantages for the production of biologics, e.g., easier isolation and purification, the absence of toxic intracellular accumulation of a heterologous protein, and hence reduced production costs [14]. Moreover, *S. cerevisiae* is resistant to high osmolality, low pH, and various inhibitors, and these properties allow for cheap and simple fermentation processes with rapid cell growth under aerobic and anaerobic conditions [15,16], which is important in industrial fermentation settings.

In spite of a number of obvious advantages of these yeasts as host cells, there are some limitations on their use as expression systems, for example, inefficient secretion, misfolding, hyperglycosylation, and aberrant proteolytic processing of some proteins [12,17,18,19,20,21,22].

Over the past decade, nontraditional yeast species have been adapted to the role of alternative hosts for the production of recombinant proteins, e.g., *Komagataella phaffii*, *Hansenula polymorpha*, *Yarrowia lipolytica*, *Schizosaccharomyces pombe*, and *Kluyveromyces lactis*. These yeast species’ several physiological characteristics (that are different from those of *S. cerevisiae*), such as faster growth on cheap carbon sources and higher secretion capacity, make them practical alternative hosts for biotechnological purposes.

Of interest to us is the yeast most widely used in this regard: *K. phaffii*. This species of yeast is successfully employed for the heterologous production of enzymes for various purposes, including those important for agriculture, thus indicating the high potential of this microorganism for industrial applications, especially in terms of secretion capacity and the ability to post-translationally modify proteins (these qualities positively affect properties of enzymes) as well as the ability to grow to high cell density. On the other hand, it should be noted that the secretion system of these yeasts has several bottlenecks that limit the yield of a recombinant protein. Among the bottlenecks, membrane translocation, signal peptide processing, and folding within the ER were suggested to be the most important limitations in terms of recombinant protein production and secretion [23], and one of the ways to overcome them is the proper choice of engineered host strains. 

Recently, yeast strain engineering through genetic modification emerged as the most helpful and effective approach to overcoming these bottlenecks. Nevertheless, advancements in this field are complicated by the presence of many cross-interacting proteins in this yeast’s secretion system; this drawback hampers genetic modification and points to the need to develop a new strategy for the genetic engineering of this system.

## 2. Specific Features of *K. phaffii* as a Producer of Heterologous Proteins

*K. phaffii* is a methylotrophic yeast from the order Saccharomycetales and is classified as a nontraditional producer [24]. It was described in the 1960s as *Pichia pastoris*, capable of utilizing methanol as a sole carbon source. In 1995, *P. pastoris* was reclassified as a member of the genus *Komagataella* [25]. The old name—*P. pastoris*—is actively used as of 2023.

Currently, the expression system based on *K. phaffii* is one of the most popular for the production of heterologous proteins due to its GRAS status, rapid growth rate on various inexpensive substrates, including, in addition to methanol, glycerin, glucose, and sucrose, as well as the ability for high-cell-density fermentation [26,27]. Along with a low secretion of endogenous proteins, *K. phaffii* efficiently and with high yields produces and secretes heterologous proteins, thereby reducing the cost of purifying the latter [28,29,30]. To date, practical approaches have been developed and technological solutions have been described for the efficient expression of recombinant proteins in *K. phaffii* [31,32].

Interest in the industrial production of proteins via *K. phaffii* for various applications—feed, food additives, detergents, waste treatment processes, and textiles—keeps on increasing [26,33]. Compared to the established baker’s yeast *S. cerevisiae*, *K. phaffii* offers a higher yield of a heterologous protein, grows to a higher cell density, can use methanol as a sole carbon source, and has certain advantages in protein maturation, including those involving protein folding and glycosylation [34]; these features have a positive effect on the properties of enzymes and on their thermal stability [35,36]. *K. phaffii*’s ability (absent in *S. cerevisiae*) to enlarge its cell biomass and hence the yield of a desired protein is due to the fact that when cultivated under aerobic conditions, *K. phaffii* does not synthesize ethanol [37].

These advantages, coupled with the observation that most of experimental methods (such as cultivation, transformation) developed for *S. cerevisiae* are easily adaptable to *K. phaffii*, make this species an ideal eukaryotic expression system [38].

### 2.1. Methods and Approaches Used to Create Strains from K. phaffii

**Figure 1:**
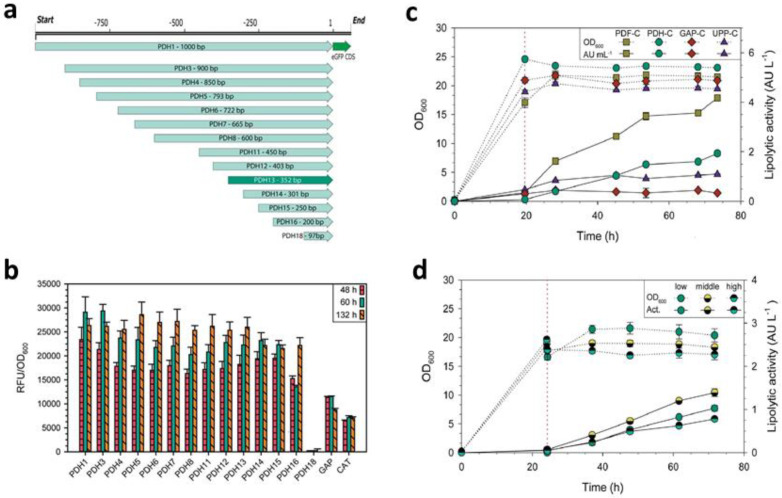
The development of methanol-free expression system based on a heat-shock gene promoter (PDH) using glycerol as sole carbon source. (**a**,**b**) The selection of the shortest putative sequence of the HPSP12 gene promoter from the *K. phaffii* genome, demonstrating the expression level comparable to that of the full-length promoter; (**c**) comparative analysis of the PDH-C promoter efficiency with the reference GAP-C, UPP-C, and PDF-C promoters; and (**d**) the impact of osmolarity levels on the efficiency of the PDH-C promoter. Adapted from [49].

To date, a wide arsenal of bioengineering techniques has been created to increase the yield of expressed and secreted proteins in *K. phaffii* [39]. The main efforts are focused on (i) gene codon optimization, which substantially raises the expression level of heterologous genes [40,41,42], (ii) increasing the gene’s copy number [43,44,45], (iii) promoter selection (Figure 1) [46,47,48,49], (iv) modification and optimization of signals of secretion and termination [50,51,52,53,54], (v) optimization of folding and of secretion pathways of the protein [55,56,57], and others (see reviews: [58,59,60]).

Figure 1 shows an example of identifying new efficient promoters by searching for highly expressed genes using RNA sequencing data and their further reconstruction to create a strictly inducible synthetic promoter for the expression of heterologous genes. Utilizing this approach, the *HPSP12* gene *from K. phaffii* was identified, and employing its promoter, a new *K. phaffii*-based heterologous gene expression system was developed. This system is strictly inducible under carbon-starving conditions using glycerol as the sole carbon source [49].

In recent years, other approaches have also been utilized to construct highly productive strains from *K. phaffii*. For instance, to introduce gene mutations in order to enhance the expression of target proteins, researchers exploit the possibilities offered by directed evolution [61,62]; biosensors are used to screen and identify highly productive clones from a heterogeneous set of transformants, including biosensors based on the fluorescent protein GFP and on the protease from the tobacco etch virus [63], and approaches are being designed to search for and isolate strains with an improved ability to secrete heterologous proteins [64]. For example, a significant improvement in the thermostability of glucoamylase from *Aspergillus awamori* X100 has been achieved [61]: a 2.7-fold increased production of cellulolytic β-glucosidase D2-BGL [62], a 1.3-fold increased xylanase activity [65], and an approximately 2-fold increased cellulase activity [66].

Considerable developments in molecular tools, including synthetic promoters for the fine-tuning of expression [67] and CRISPR/Cas9 technology [68,69], have led to the creation of highly secreting strains with the participation of *K. phaffii* as a host [70,71].

Let us examine in more detail vector expression systems (including promoters, selection markers, and secretion signals)—the choice of which is a crucial prerequisite for the successful production of a recombinant protein by *K. phaffii*—as well as the main factors influencing the productivity of strains.

#### 2.1.1. Promoters for the Expression of Heterologous Genes in *K. phaffii*

To this end, two types of promoters are used: inducible and constitutive, each of which has its own advantages and disadvantages [72,73]. For instance, when inducible promoters are employed, it becomes possible to separate the phase of culture growth and accumulation of cell biomass from the phase of a target protein’s production; this separation reduces cellular stress during cell growth and creates conditions for good production of proteins, including those toxic to *K. phaffii*. This strategy is especially suitable when the co-expression of auxiliary proteins, such as chaperones, is necessary before the synthesis of the target protein begins to prevent cellular stress during the production of the target protein. When constitutive promoters are utilized, cultivation is facilitated, which in this case does not require a change of the carbon source, and the product is quickly synthesized; however, the high metabolic load on the cells in their growth phase will reduce their viability and is not suitable for the production of proteins toxic to yeast cells [72,73]. To date, a number of studies and reviews are available about using different promoters for heterologous expression, e.g., this study [32].

Among the promoters most commonly used for the expression of recombinant proteins in *K. phaffii*, it is worth mentioning the tightly regulated methanol-inducible promoter of the alcohol oxidase I gene (pAOX1) and the strong constitutive promoter of the glyceraldehyde-3-phosphate dehydrogenase gene (pGAP) [74,75]. pGAP is used in cells that can be cultured not only on glucose and glycerol but also on methanol; therefore, this promoter can be employed together with pAOX1 to increase the production of a target protein [47,76]. The promoter pFLD1 of glutathione-dependent formaldehyde dehydrogenase 1 is being investigated as an attractive alternative to pAOX1, and pFLD1 is strongly and independently induced either by using methanol as the sole carbon source or by using methylamine as the sole nitrogen source [77,78].

**Figure 2:**
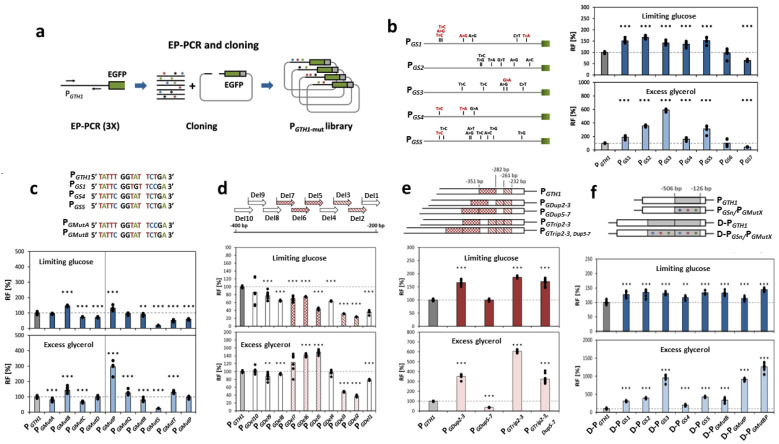
The development of methanol-free expression system based on the glucose transporter gene promoter (pGTH1) [83]. (**a**) Schematic of the workflow for generating a *K. phaffii* GTH1 promoter library by random mutagenesis; (**b**,**c**) impact of single point mutations on expression properties of PGTH1: (**b**) random mutagenesis (the red colored mutations are located outside of a transcription factor binding sites), (**c**) systematic introduction of single point mutations into the transcription factor binding site; (**d**) impact of the segmental deletions introduced to the −200 to −400 bp region of pGTH1 on its expression properties; (**e**) impact of the segmental duplications on expression properties of pGTH1; and (**f**) impact of the duplication of the main regulatory promoter region and point mutations on expression properties of pGTH1. Designations: Bars represent mean values and closed circles the calculated RF values of the individual clones; the horizontal dotted line highlights the average expression level of the native PGTH1 control strain (set to 100% for each condition); **—*p* ≤ 0.05; ***—*p* ≤ 0.005. Adapted from [83].

There are other known strong promoters that are utilized for the synthesis of heterologous proteins in *K. phaffii*. Among the constitutive ones, it is worth noting the promoter of the *TEF1* gene of the translation elongation factor 1 (pTEF1), which is comparable to pGAP, and the promoter of the *GCW14* gene (pGCW14), which is stronger than pGAP and pTEF1 [79,80], as well as new promising regulated promoters based on the promoter pCAT1 of the catalase gene [81], on pSDH (from the sorbitol dehydrogenase gene) [82], or on pGTH1 (from the glucose transporter gene) (Figure 2) [83], as well as bidirectional promoters: histone promoters and synthetic hybrid ones [84,85].

Figure 2 presents an example of a comprehensive approach to the rational design of the GTH1 glucose transporter gene promoter (pGTH1), which enabled the development of an efficient system for expressing heterologous proteins in *K. phaffii* based on this promoter, strictly inducible under glucose limitation [83]. The approach includes random mutagenesis (Figure 2a,b) and targeted engineering of the promoter sequence using point mutations (Figure 2c), deletions (Figure 2d), duplications (Figure 2e), and combinations of duplications and mutations (Figure 2f).

Considering pAOX1’s strict methanol dependence, which limits its applications (especially in industrial production), new, methanol-free systems for the induction of pAOX1 have been designed [86], including the one based on the reconstruction of a synthetic positive feedback loop controlled by transcription factor Mxr1; this system has turned out to be even more effective than methanol-based ones [87].

#### 2.1.2. The Choice of a Signal Sequence

For secreted proteins, signal peptides are the sorting signal that directs proteins from the cytosol into the extracellular milieu, and the efficiency of signal peptides directly affects the release of target proteins into the culture medium. In *K. phaffii*, various signal sequences are employed to secrete target proteins. There are known examples of the use of signal sequences from native genes of acid phosphatase and α-amylase of *K. phaffii*, from the *SUC2* gene of *S. cerevisiae*, from the bovine β-casein gene, or from the *nsB* gene of lipase B of *Candida antarctica* [88,89,90,91,92,93,94] as well as synthetic peptides (MF4I) [40].

Nonetheless, one of the most popular signal sequences for target protein secretion in *K. phaffii* is the sequence of the α-factor (α-MF) from *S. cerevisiae* [95]. It consists of a pre- and pro-region: the former is responsible for the post-translational routing of a synthesized protein into the endoplasmic reticulum (ER), and the latter presumably participates in the transfer of a protein from the ER into the Golgi apparatus. The processing of α-MF in the course of protein secretion is carried out in three steps: the first one is the removal of the pre-region by signal peptidase in the ER, and then the pro-region is processed in two steps: cleavage by the endoprotease Kex2 at amino acid site Lys-Arg and cleavage with the help of the Ste13 protein in the region of Glu-Ala, which repeats the target protein inside the Golgi apparatus [96,97].

One of the common problems with using the signal sequence of the α-MF is the occurrence of nonfunctional types of product caused by the N-terminal extension of recombinant proteins owing to the incomplete processing of the signal peptide; the problem has been solved by a modification of the peptide’s N terminus to improve its steric accessibility to proteases [98].

To enhance the potential of the α-MF, other strategies have been applied too, including codon optimization, directed evolution [99], spacer insertion, and deletion mutagenesis [97]. For example, mutants of the α-MF—where at position 42, amino acid residue Leu is replaced by Ser, and at position 83, Asp is substituted with Glu—increase the productivity of the recombinant protein by almost threefold as compared with the wild type [52]. Deletions in the α-MF pro-region (Δ57–70) have improved the efficiency of expression of some heterologous genes in *K. phaffii* cells by almost an order of magnitude [100,101,102].

There is another limitation that negatively affects protein secretion when the *S. cerevisiae* α-MF is applied. Unfortunately, the α-MF causes a post-translational translocation of its protein through the ER membrane [103]. If the α-MF is fused to a protein that can fold in the yeast cytosol, then the translocation of such a protein across the ER membrane and consequent secretion will be ineffective. One of the solutions involves a chimeric signal sequence in which the *S. cerevisiae* pre-α-MF is replaced with the corresponding region of *S. cerevisiae* Ost1, which facilitates a cotranslational translocation of the protein across the ER membrane and ensures that protein folding occurs only after the ER lumen is reached [104]. Indeed, such a replacement has considerably increased the efficiency of secretion and production of various heterologous proteins [52,105]. This mechanism of cotranslational translocation can be helpful in the case of hydrophobic proteins, which will be immediately sent into the ER lumen for further transport, without accumulating in the cytoplasm [104].

One of the research fields actively advancing in the last decade is the search for new sequences with a high secretion capacity. For instance, analyses of transcriptomic and proteomic data have identified in *K. phaffii* a signal peptide called PAS_chr3_0030, which has successfully been utilized to secrete the NIT10 enzyme, which failed to be secreted when the α-factor was used [106]. Screening of the endogenous signal peptides in *K. phaffii* has revealed a series of signal sequences (Scw, Dse, Exg, Dan4, Gas1, Msb2, Fre2, and others) that are comparable or even superior to the α-MF from *S. cerevisiae* in the production of heterologous proteins [107,108,109,110]. A similar analysis of α-MF signal peptides from various yeast species has shown that the efficiency of protein secretion when the α-MF from *K. lactis* is employed is comparable to that from *S. cerevisiae*, whereas with the α-MF from *Wickerhamomyces ciferrii*, the efficiency is even higher [54,110]. All these sequences can potentially be used instead of the *S. cerevisiae* α-MF to secrete heterologous proteins in *K. phaffii*.

#### 2.1.3. Specifics of Introduction of Foreign DNA into *K. phaffii*

To introduce a target gene into *K. phaffii* cells, various vectors are employed. The standard vectors for the heterologous expression of target proteins represent a bifunctional system that is capable of replicating in *E. coli* and can be maintained in *K. phaffii* by means of auxotrophy markers (such as HIS4, MET2, ADE1, ARG4, URA3, URA5, and GUT1) or genes of resistance to antibiotics such as zeocin, geneticin, and blasticidin [111,112,113]. Strains possessing resistance to antibiotics, as a rule, cannot be used for such production; therefore, after a strain is selected, antibiotic resistance genes are removed most often by means of the Cre-lox recombination system, which allows an investigator to eliminate such a gene while preserving the DNA region containing the target gene [114].

Episomal plasmids typically are not utilized for heterologous expression in *K. phaffii*. The most preferred method is the integration of a linearized expression cassette into the genome of the host cell via homologous recombination. In *K. phaffii* cells, however, nonhomologous end joining often takes place instead. To increase the chances of homologous recombination, longer homologous flanking sequences are used, which reduce the efficiency of nonhomologous end joining [115].

When a vector with pAOX1 is employed, the site mediating homologous recombination is a region of this promoter. To improve the efficiency of the integration of plasmid DNA into the *K. phaffii* genome, a cloned vector is linearized with the help of such restriction endonucleases as *Sac*I, *Pme*I, or *Bst*XI. When such a vector is integrated into the cell genome through homologous recombination, only a single insertion occurs in most cases, but in 1–10% of cases, multiple insertions take place [116].

#### 2.1.4. An Increase in the Copy Number of a Target Gene as a Way to Promote Protein Production

One of the approaches commonly applied to enhance the production of recombinant proteins is to increase the copy number of a reporter gene through multiple transformation procedures and post-transformation vector amplification [117,118].

A similar result can be obtained by direct selection: the transformation of cells with an expression vector carrying an antibiotic resistance marker, followed by the selection of multicopy clones at increasing concentrations of the antibiotic. In this way, a *K. phaffii* strain has been created having multiple (eight) copies of the α-amylase gene under the control of the *AOX* promoter; this strain produces an order of magnitude more of a recombinant protein as compared to a single-gene-copy clone [75].

Alternatively, defective auxotrophic markers can be used to increase the copy number of a reporter gene. Typically, these markers are transcriptionally deregulated genes that lack most of the promoter region. Cells transformed with this marker can restore prototrophy by increasing the number of copies of the defective marker. Amplification of the defective marker gene is accompanied by the amplification of a neighboring heterologous gene [119]. An example of such a defective marker is *leu2-d*: a *LEU2* allele that takes part in leucine metabolism [120] and is commonly employed in *S. cerevisiae* to maintain a high copy number of a plasmid under selective pressure [121]. A similar system has been developed for *K. phaffii* [122].

The advantage of post-transformation vector amplification lies in its technical simplicity; however, among its disadvantages, one can list the probabilistic nature of copy number increase and the high consumption of antibiotics when using an antibiotic resistance gene as a selection marker.

Another popular method is the use of vectors carrying multiple copies of a target gene together with selection markers. Subsequently, the antibiotic resistance gene is removed by means of the Cre-lox system, and it becomes possible to re-transform the resultant strain to raise the target gene’s copy number, which makes this recombination system a powerful tool for the multiple integration of target genes. With the help of this system, highly efficient expression vectors have been constructed for generating multicopy transformants in *K. phaffii* that significantly strengthen the synthesis of a target protein [123].

The advantage of this method is the controlled successive increase in the copy number of the target protein gene. However, a drawback is the need to remove selection markers after each cycle of copy amplification.

### 2.2. Bottlenecks of Secretion in K. phaffii: The Unfolded Protein Response (UPR) and Vacuolar Degradation of Heterologous Proteins

As mentioned above, in the yeast *K. phaffii*, the secretion system has bottlenecks that limit the release of a recombinant protein. It has long been known that one of these bottlenecks is the low rate of processing of heterologous proteins in the ER, resulting in the accumulation of unfolded proteins within the cell and in incorrect processing and folding of complicated recombinant proteins (see reviews: [34,124,125,126]. Because only correctly folded proteins can be secreted, the accumulation of unfolded or incorrectly processed proteins in the ER induces a stress response aimed at their elimination. This is so-called ER stress, which, on the one hand, activates a cell-protective program (UPR) [127], leading to the upregulation of genes whose products promote the correct assembly and processing of proteins, including the transcription factor Hac1 (the activator of this response), chaperone Kar2, and modification enzymes (protein disulfide isomerase Pdi1 and disulfide oxidase Ero1) [13,57,128], and on the other hand, activates the ER-associated pathways for the degradation of improperly processed proteins [129,130]. Factors contributing to the initiation of a stress response in *K. phaffii* are intensive synthesis of recombinant proteins, especially membrane ones or complex secreted proteins, and unsuitable cultivation conditions [131,132,133,134,135].

The key proteins from this section, their functions, and examples of co-overexpression are presented in Table 1 for a comprehensive overview.

In this regard, the most common approach to enhancing the secretion of heterologous proteins is the co-expression of target genes with the genes of chaperones, which facilitate the correct folding of recombinant proteins [57]. Indeed, it has been demonstrated that the overexpression of genes encoding key components of the UPR (including genes of chaperone Kar2 and of modifying enzymes Pdi1 and Ero1) increases the production of heterologous proteins when *K. phaffii* serves as a cell host [23,136,138]. Besides, it is reported that in *K. phaffii*, a similar effect on the secretion of a heterologous protein is exerted by a co-overexpression of the genes of chaperones SSA4 and SSE1, the genes *BMH2* and *BFR2* of the proteins involved in transport, and the genes *Sec1* and *Sly1* of the proteins participating in the transport of vesicles as well as the gene of the protein kinase KIN2, which is associated with exocytosis, and the gene of the CUP5 subunit of the vacuolar ATPase [23,136]. As for the activator of the UPR (transcription factor Hac1), its influence depends on a given heterologous protein: the overexpression of Hac1 can increase, decrease, or have no effect on the synthesis of the target protein [140].

At the same time, there is evidence that the co-expression of target genes with the genes of chaperones and of modification enzymes can affect not only the secretion of recombinant proteins but also the proliferation rate of *K. phaffii* strains, which also influences their productivity. Furthermore, this phenomenon is ambiguous. For instance, it has been found that the co-expression of the *secA* gene of cephalosporin C-acylase with genes *Hac1*, *Kar2*, *Pdi2*, *Mpd1*, and *Sil1* of the UPR and their folding factors significantly diminishes the proliferation rate of modified *K. phaffii* strains [109]. The reduction in the proliferation rate of the cultured yeast is also observed with the co-expression of the gene of Gal β-galactosidase with the gene of chaperone Kar2 [109], while in other articles, researchers have not noticed a negative impact of *Ero1*, *Kar2*, *Pdi1*, *Sec1*, and *Sly1* overexpression on the growth of cells secreting the recombinant fusion protein IL2-HSA [23]. Additionally, the above phenomenon was absent during the co-expression of the *secA* gene of cephalosporin C-acylase and of the A33scFv fragment of the single-chain antibody A33 with the *Pdi1* gene of disulfide isomerase [107,141]. There are examples of a positive influence of UPR genes on the growth of *K. phaffii* cells; in particular, such an effect is seen after the co-expression of the *rPAE* gene of elastase from *Pseudomonas aeruginosa* with the *Hac1* gene [142]. These data indicate that for the correct assessment of the impact of a co-expressed helper gene on the production/secretion of a heterologous protein, it is important both to compare concentrations or activities of the proteins and to compare growth characteristics of the strain, which are specific to each heterologous protein and determine the final yield of the product.

Another aspect of the response to ER stress substantially affects the target protein’s production: heterologous proteins’ vacuolar degradation, associated with the mis-sorting of recombinant proteins and their transport to a vacuole, where the proteins are broken down and recycled [143]. It has been shown that mutations in subunits of a complex called CORVET (core vacuole/endosome tethering, class C, which functions at early stages of endosomal sorting), in combination with a knockout of vacuolar proteases, promote the secretion of heterologous proteins [144]. A similar result has been obtained via a combination of mutations in the *vps* (vacuolar protein sorting) genes of the HOPS complex (homotypic fusion and protein sorting, involved in the fusion of late endosomes with lysosomes), with a knockout of vacuolar proteases Pep4 and Vps70 [145]. An even more pronounced influence on the secretion of a heterologous protein in mutant strains in genes of the HOPS complex is exerted by the overexpression of the *Sbh1* gene encoding a subunit of the ER translocation pore [145]. These findings suggest that in *K. phaffii*, vacuolar degradation is one of the predominant pathways for the degradation of the recombinant proteins that for various reasons cannot get translocated and get stuck on the cytosolic side of the translocation channel [145], and the disruption of these complexes facilitates the secretion of these proteins.

It was stated above that unsuitable cultivation conditions [131] can also provoke a stress response lowering the secretion of heterologous proteins. Besides, this situation occurs after switching from a shake flask low-density culture to a high-density culture in bioreactors; accordingly, one important strategy for increasing the final yield of recombinant proteins during industrial production is a considerable re-optimization of such culture conditions as pH, aeration, and the feed rate of a carbon source [146].

In conclusion of this section, it should be added that despite some conservatism of the system of the response to ER stress in eukaryotes [127,133], the molecular-genetic and physiological aspects of the development of this response differ among yeast species [147]. This is true for *K. phaffii* too, which differs appreciably from *S. cerevisiae* both in the level of basal stress and in specific mechanisms underlying the progression of the UPR [57,140,148,149]. This state of affairs does not allow us, without additional basic research, to use the data obtained in *S. cerevisiae* to devise ways of enhancing the productivity of *K. phaffii* strains.

### 2.3. Distinctive Features of Post-Translational Modifications in K. phaffii and Their Impact on the Production of a Heterologous Protein

The most common post-translational modifications of proteins in eukaryotes are their glycosylation and the formation of disulfide bonds, which affect proper protein folding. As mentioned above, in *K. phaffii*, the incorrect folding of heterologous proteins provokes the stress response that reduces the magnitude of their secretion. Possible ways to influence these processes are some of the important aspects of metabolic engineering when microbial strains are designed that produce recombinant proteins. The ability to glycosylate proteins and form disulfide bonds is the greatest advantage of *K. phaffii* over bacterial systems.

#### 2.3.1. Introduction of Disulfide Bonds

*K. phaffii* is the most commonly used yeast species for the production of proteins rich in disulfide bonds (S–S) and requiring post-translational modifications [150]. The enzyme that creates such bonds under the oxidative conditions of the ER is protein disulfide isomerase PDI [151], which has a complicated domain structure [152], enabling not only the catalysis underlying the formation of S–S bonds but also a reversible interaction with various peptides and misfolded proteins [153].

In the yeast *K. phaffii*, the overexpression of PDI is reported to amplify the secretion of both recombinant proteins rich in disulfide bonds [154,155,156] and proteins lacking thereof [157]. This evidence confirms the finding that protein disulfide isomerase PDI is an important molecular chaperone as well [158], whose catalytic activity is most pronounced toward partially unfolded and misfolded proteins [153].

#### 2.3.2. Glycosylation

Many proteins are glycoproteins and require the attachment of carbohydrate structures to the protein backbone (glycosylation) to ensure the proper folding, solubility, stability, and proper biological activity of a protein [146]. In *K. phaffii* (as in other yeasts), there are two main modes for the glycosylation of proteins: N- and O-glycosylation. N-glycosylation is mediated by the amino group of an asparagine residue, giving rise to an amide bond, whereas in O-glycosylation, the oxygen in the side chain of serine or threonine is linked to a carbohydrate moiety via an ester bond [159]. Both types of modification are initiated in the ER and affect the dynamics of secretion of recombinant proteins [160,161,162,163]. N-glycosylation plays an important role in the folding and quality control of glycosylated proteins, while O-glycosylation is crucial for protection from proteolysis [29,164]. Both types of glycosylation in yeasts differ from these processes in higher eukaryotes, including humans; this is because typical yeast N- and O-glycans contain chains of mannose residues, which—in the case of N-glycans—are adjacent to two N-acetylglucosamine (GlcNAc) residues, whereas in the case of O-glycans, the mannose residues are adjacent to serine or threonine residues; by contrast, proteins of higher eukaryotes contain so-called complex N-glycans consisting of GlcNAc, galactose, and sialic acid [29,165].

**Figure 3:**
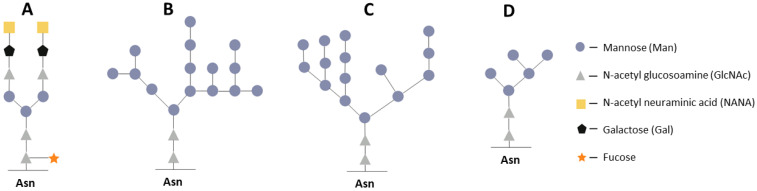
Schematic diagram of N-linked glycan structure in a mammalian cell, *S. cerevisiae*, and *K. phaffii*. (**A**) N-linked glycan structure in mammalian cells commonly generates complex terminally sialylated structures. (**B**) In *S. cerevisiae*, the N-linked glycan structure is typically hypermannosylated (Man > 50GlcNAc2). (**C**) N-linked glycan structure in *K. phaffii* typically is of the Man8-14GlcNAc2 type with a triantennary-branched structure. (**D**) In Pichia GlycoSwitch^®^ strains (SuperMan5), N-linked glycan structure is typically hypomannosylated (with a mannose-5 structure). Adapted from [29].

It is the presence of a large number of mannose residues in yeast glycans that is one of the factors contributing to the hyperglycosylation of heterologous proteins, their incorrect processing, and their elevated immunogenicity [29,166]. Nonetheless, in *K. phaffii*, in contrast to *S. cerevisiae*, glycosylation is less intense due to shorter chains of N-linked oligosaccharides (8–20 vs. 50–150 units), and the N-glycans of *K. phaffii* do not contain immunogenic terminal α-1,3-linked mannose residues because of the absence of the Mnn1 enzyme needed for this modification [13,163,167]. Instead, *K. phaffii* possesses Bmt family enzymes, which catalyze the addition of β-1,2 mannose. Furthermore, *K. phaffii* has low levels of O-glycosylation [168,169]. A comparison of the structures of N-linked glycan in a mammalian cell, *S. cerevisiae*, and *K. phaffii* is shown in Figure 3.

The above-mentioned features of the glycosylation system of *K. phaffii* make this yeast species more attractive as a producer of heterologous proteins in comparison with *S. cerevisiae*. Nevertheless, issues of optimization of glycosylation processes, when strains producing certain recombinant proteins are created on the *K. phaffii* platform, remain some of the important topics in metabolic engineering [165,166,170,171,172,173]. It should be noted that, using glycoengineering approaches, a number of encouraging results have been obtained (for example, increased solubility, stability, and changes in pharmacokinetics) [163].

In conclusion of this section, we should point out that specific features of the expression of heterologous proteins by means of *K. phaffii* and the usefulness of this yeast as a cellular factory for the production of recombinant proteins are also discussed in other reviews [95,111,112,113,174,175].

As for the application of *K. phaffii* in obtaining the enzymes that can serve as feed additives in animal husbandry, we will next examine the enzymes that are most needed in agriculture and the *K. phaffii* strains that produce them.

## 3. Enzyme-Based Supplements for Preparation of Feeds

Today in agriculture, the most in-demand enzymes are phytases, amylases, and glucoamylases, which hydrolyze phytate and grain starch, as well as xylanases, mannanases, and cellulases, which catalyze the hydrolysis of nonstarch polysaccharides (xylans, mannans, β-glucans, and cellulose) and the proteases that hydrolyze plant proteins, which constitute a considerable proportion of feed by weight but have low digestibility (%).

The improvement of the nutritional value of feeds—and the associated increase in animal productivity—is not the only reason for interest in these enzymes in the field of agriculture. For instance, the low digestibility of some starches is involved in the onset of some gastrointestinal diseases because undigested and unabsorbed starch, when entering the large intestine, can serve as a substrate for bacterial fermentation, promoting the proliferation of some potentially dangerous pathogenic bacteria [176]. Aside from improving the nutritional value of feeds, the use of certain enzymes in animal nutrition (for example, laccase as a food additive for feeding broiler chickens) substantially reduces the residual level of antibiotics in broiler droppings and alleviates the dysbiosis of the intestinal microbiota when antibiotics are overused [177]. Cellulases also have a positive impact on cecal fermentation processes by enhancing the production of propionic acid, which acts as a bacteriostatic agent and thereby can diminish colonization by pathogenic bacteria [176].

It is widely accepted that thermostable and acidophilic enzymes are desirable in feed additives because good thermal stability allows the enzyme to withstand the high temperatures required for the feed-pelleting procedure, whereas resistance to an acidic medium (pH 1.5–3.5) present in the digestive tract of monogastric animals helps to preserve high enzymatic activity.

According to a Global Animal Feed Enzymes Market Report, the global volume of feed enzymes reached USD 1340.6 million in 2021 and is expected to grow at a compound annual growth rate of 5.0% during the 2022–2028 period (cited by [178]). These statistics indicate that enzymes are becoming an important ingredient in feed manufacture.

At present, approved feed enzymes are ubiquitous in animal feed, are nontoxic, and after consumption, are easily broken down into amino acids that are indistinguishable from the amino acids of other food sources; consequently, the main determinant of the safety of an enzyme for feed supplementation is the producer microorganism [179]. These are yeast *K. phaffii*, which are extensively used as an efficient platform for heterologous recombinant protein production due to its GRAS status, rapid growth rate, and ability for high-cell-density fermentation [29].

## 4. *K. phaffii* as a Producer of Enzymes for Increasing Nutritional Value of Feed

Today, the expression system based on *K. phaffii* is one of the most popular for the production of heterologous proteins due to the growth and metabolic features of this yeast described above. The yeast *K. phaffii* is successfully utilized for the heterologous production of enzymes important for the feed industry. The most complete list of these enzymes is given in the review [26]. Let’s consider the most widely used, among them are phytases, amylases, and glucoamylases, which hydrolyze phytate and grain starch, as well as xylanases, mannanases, and cellulases, which catalyze the hydrolysis of nonstarch polysaccharides (xylans, mannans, β-glucans, and cellulose) and the proteases that hydrolyze plant proteins.

The following are examples illustrating the use of genes from certain organisms the for heterologous production of these enzymes by means of *K. phaffii*.

### 4.1. K. phaffii as a Producer of Phosphohydrolytic Enzymes 

#### Phytase

Phytase (myo-inositol hexakisphosphate phosphohydrolase) is an enzyme that hydrolyzes the phosphoester bonds of phytates with a gradual release of inorganic phosphates and myo-inositol phosphate derivatives. This enzyme is most commonly added to pet foods [180]. This is because pig and poultry diets are typically based on grains and oil crops, where up to 70–80% of the phosphorus content is bonded to phytate (a phytic acid salt, myo-inositol hexaphosphate) [181]. Phytate is poorly utilized during digestion by these animals owing to the absence of an endogenous phytase activity. Exogenous phytase not only increases the availability of phosphorus to the animal but also betters the digestibility of other key nutrients [179,180,181]. The bioefficacy of supplemental phytase in pig and poultry diets is well established [182,183,184,185,186,187].

Phytases were discovered by Suzuki et al. in 1907 in rice bran (cited by [188]). They are widespread in nature and can be synthesized by a variety of organisms, including microorganisms, plants, and animals [189]. 

The focus of research attention so far has been on phytases derived from filamentous fungi, including *Aspergillus*, *Mucor*, *Penicillium*, *Rhizopus*, and others [190,191,192,193,194], among which, *Aspergillus ficuum* NRRL 3135 has been identified as the most active natural producer of phytase [195]; the phytase isolated from *Citrobacter braakii* YH-15 has the highest specific activity among known phytases and is almost twice as active as *E. coli* phytase [196], phytase isolated from *Aspergillus niger* UFV-1 has characteristics that make it promising for industrial use, including the manufacture of feed additives. This enzyme shows maximal activity at pH 2.0, retains more than 90% of its activity at 60 °C for 120 h, and has manifested a strong resistance to pepsin and trypsin [191]. Today, approved feed phytases are manufactured using fungal or bacterial genes from *Aspergillus niger*, *E. coli*, *Citrobacter braakii, Buttiauxella* spp., and others [194,197,198]. 

In the literature, information about the construction of strains of *K. phaffii* for the production of phytase mainly deals with research on the expression of the *appA* phytase gene of *E. coli* in the yeast *K. phaffii* under the control of the strong constitutive promoter pGAP of the gene of glyceraldehyde-3-phosphate dehydrogenase and promoter pAOX1 of the alcohol oxidase 1 gene, inducible by methanol [43,51,199,200,201]. 

It is important to add that in the case of the appA gene from *E. coli*, the inducible expression system of *K. phaffii* was found to be three times more efficient than constitutive expression, 40 times more efficient than the expression system of *Saccharomyces cerevisiae*, and two orders of magnitude more efficient than the expression system of *Schizosaccharomyces pombe* [202].

Recently, genes of phytase from other organisms have been identified, the expression of which in *K. phaffii* has allowed for the production of highly active acidic phytase preparations. Moreover, the stability of some phytases, including AppA from *E. coli*, at high temperatures was significantly higher when *K. phaffii* was used as an expression platform compared to *E. coli* [198,203], partly due to the ability to optimize glycosylation sites on the phytase. For example, optimization of the number and localization of glycosylation sites of phytase from *Yersinia intermedia* produced by *K. phafii* GS115 increased the protein half-life from 3.32 min at 65 °C to 25 min at 100 °C [204].

The characteristics of phytase preparations obtained using *K. phaffii* as an expression system, with a high acid pH and high-temperature stability, as well as tolerance to pepsin and trypsin, are provided in Table 2.

From the data presented in Table 2, it can be concluded that to date, phytase preparations obtained with *K. phaffii* expression system have shown significantly higher activity compared to industrial phytase from *A. niger* (100 U/mg; [209]), and is also higher than or comparable to the activity of a commercial phytase from *Peniophora lycii* (864 U/mg; [210]).

The data presented above indicate the possible suitability of *K. phaffii* for the production of highly active thermostable phytases, at a minimum by means of bacterial phytase genes from *E. coli* or *Citrobacter* spp., which are appropriate sources of phytase supplements for feed manufacture [211].

### 4.2. K. phaffii as a Producer of Enzymes That Hydrolyze Starch

#### 4.2.1. α-Amylase

α-Amylase (α-1,4-glucan-4-glucanohydrolase) is an endoamylase, and it catalyzes the hydrolysis of α-1,4-glycosidic bonds within the chain of starch and related carbohydrates, thereby generating substances with a low degree of polymerization, such as glucose, maltodextrin, and oligosaccharides of various lengths.

α-Amylase is widely employed in various industrial applications [212,213], including feed manufacture [178]. Currently, thermostable α-amylases—which are important for technological processes involving high temperatures, including the production of animal feed—are in demand. The most common sources of thermostable α-amylase are bacteria isolated from hot springs. These amylases have greater structural flexibility than mesophilic α-amylases [214]. 

Research on α-amylase production is linked with a search for strains synthesizing highly efficient enzymes, and the improvement of various characteristics of the synthesis, such as folding (overexpression of chaperones) and secretion of recombinant proteins (optimization of signal peptides), protection from protease degradation (repression of endogenous protease genes), and other topics [215,216,217], as well as a search for inexpensive substrates for the industrial cultivation of producer strains [218]. 

Microbe-derived α-amylases have attracted attention due to their low production costs, stable fermentation, and short production cycle [219]. α-Amylases from bacterial species *Bacillus subtilis*, *B. amyloliquefaciens*, *B. licheniformis*, and *B. stearothermophilus* are industrially significant [220].

At present, the commercial production of α-amylases via microorganisms such as fungi, yeasts, and bacteria constitutes approximately 30% of the global enzyme market [221]. It is estimated that by the end of 2024, the global α-amylase market will reach USD 320.1 million [218]. An analysis of patents related to practical applications of α-amylases indicates that of the 186 patents filed in the last five years [222], 84 are related to biofuel manufacturing, 41 to the production of beverages, 21 to food products and animal feed, 16 to pharmaceuticals, 15 to detergents, and nine are related to textiles. Furthermore, it has turned out that in the beverage and animal feed sectors, a preference is given to thermostable α-amylases.

Examples of recombinant thermostable α-amylases successfully cloned in *K. phaffii* are as follows: α-amylase from *Geobacillus stearothermophilus,* with a specific activity of 151.8 U/mg, a maximal activity at 65 °C, and a half-life of 88 min at 60 °C [223]; α-amylase from *Geobacillus* sp. 4j and the thermostable α-amylase encoded by genes from the bacterium *Bacillus licheniformis* were produced by *K. phaffii* with activity of 2200 U/mL [224] and 900 U/mL [225], respectively.

The α-amylase gene from the thermophilic fungus Thermomyces dupontii L18 has also been successfully overexpressed in *K. phaffii* [226]. The highest α-amylase activity (38,314 U/mL) in that work was obtained at a protein concentration of 28.7 mg/mL after 168 h fermentation. The enzyme manifested maximal activity at 60 °C and pH 6.5 and was thermostable up to 55 °C in the pH range of 4.5–10.0 [226].

A comparative analysis of characteristics of α-amylases from the bacterium *Alkalimonas amylolytica*, when synthesized using *E. coli* BL21 and *P. pastoris* GS115 as a host, detected no differences in catalytic characteristics and thermal stability between the recombinant enzymes [227]. Nonetheless, when the recombinant strain of *P. pastoris* GS115 was cultivated under optimal conditions in a 3 L bioreactor, the extracellular α-amylase activity reached 600 U/mL, which was approximately 10 times higher than that obtained during the cultivation of the *E. coli* strain [227]. These results suggest that the preferred host for α-amylase production is *K. phaffii*.

#### 4.2.2. Glucoamylase

Glucoamylase (α-1,4-glucan glucohydrolase) hydrolyzes α-1,4 and α-1,6 glycosidic bonds, sequentially cleaving off terminal α-D-glucose residues from the termini of amylose and amylopectin; in other words, this enzyme is an exoamylase. Glucoamylase is intended to saccharify partially degraded starch polymers with the formation of glucose.

Glucoamylase is produced mainly on the basis of fungal genes because it is almost absent among bacteria. Enzymes from *Aspergillus*, *Rhizopus*, and *Endomyces* dominate the market, but the former is a favorite because enzymes synthesized by it are more thermostable. During the production of glucoisomerase from the fungus *Alternaria alternata* using inexpensive industrial raw materials (dried potato powder) through solid-phase fermentation, an enzyme preparation with a specific activity of 39,200 U/g was obtained, which can be used for the commercial synthesis of glucoamylase [228]. The recombinant strains producing thermostable glucoamylases described in the literature have been designed mainly on the basis of the yeast *P. pastoris* (*K. phaffii*) [229,230,231,232,233,234]. A possible reason is the structural features of fungal glucoamylases, whose genes have been mainly used for such heterologous expression, as well as specific features of the *K. phaffii*-based expression system.

By means of the glucoamylase gene (cloned in *K. phaffii*, *S. cerevisiae*, and *A. niger*) from *Aspergillus awamori* as an example [235], it has been demonstrated that catalytic characteristics of the recombinant enzymes produced by different hosts are virtually the same; however, the glucoamylase synthesized by *K. phaffii* has manifested higher thermostability than the enzymes secreted by *S. cerevisiae* and *A. niger*, and this advantage can be explained by a change in the degree of the enzyme’s glycosylation.

Examples of recombinant thermostable glucoamylases successfully cloned in *K. phaffii* are as follows: the glucoamylase from the fungus *Aspergillus flavus* NSH9—this enzyme retains 50% of its activity when incubated for 20 min at 90 and 100 °C [232]; the glucoamylase from the soil fungus *Chaetomium thermophilum*—this enzyme retains 80% of its activity after 60 min incubation at 70 °C [229]; glucoamylases encoded by genes *TlGa15A* [234] and *TlGa15B* [213] from the fungus *Talaromyces leycettanus*—these enzymes show exceptional thermostability at 65 °C [233,234]; the glucoamylase from the ascomycete *Bispora* sp. MEY-1—the enzyme was produced by *K. phaffii* with a high yield of 34.1 U/mL and proved to be stable across a wide pH range (2.2–11.0) and at high temperatures, up to 70 °C [231]. 

There are also examples of the successful co-expression of glucoamylase and α-amylase genes from the fungus *Rhizomucor pusillus* in *K. phaffii*, resulting in higher enzymatic activity. For instance, during their separate expression, the activity of recombinant glucoamylase was 1237 U/mL, and α-amylase activity was 2927 U/mL, whereas during co-expression, these numbers were 2218 and 8285 U/mL, respectively [230]. Both enzymes were stable over a wide pH range of 4.0–9.0, and after incubation for 30 min at 60 °C, the glucoamylase retained 73% of its activity, and the α-amylase retained 85%.

Overall, we can conclude that the use of *K. phaffii* as a host for the production of glucoamylases and α-amylases is preferable for the expression of not only fungal glucoamylase genes but also bacterial α-amylase genes. Additionally, joint expression of their genes in *K. phaffii* holds promise for obtaining higher activity of the resultant glucoamylase and α-amylase.

### 4.3. K. phaffii as a Producer of Enzymes That Hydrolyze Nonstarch Polysaccharides

As for nonstarch polysaccharides (cellulose, xylans, mannans, β-glucans, and complex pectins)—which make up a substantial proportion of carbohydrates in feed by weight but have low digestibility (%)—and regarding plant proteins, there are some opportunities for enhancing their assimilation by means of enzymes synthesized in *K. phaffii*, as we will describe below.

#### 4.3.1. Xylanase

Xylanase (endo-1,4-β-xylanase) catalyzes the hydrolysis of xylan (a linear polysaccharide) into xylose, thereby degrading hemicellulose, one of the major components of the plant cell wall. Xylan is the most abundant hemicellulose in lignocellulosic biomasses among other hemicelluloses such as glucuronoxylan, arabinoxylan, glucomannan, and xyloglucan. Xylans are widespread among all types of lignocellulosic biomasses, including wood, grasses, and cereals, and account for up to 35% of their dry weight. Xylanase is widely used in the poultry industry owing to an ability to degrade arabinoxylans found in wheat and corn, which are the predominant feed grains in broiler diets [236]. Xylanase improves broiler growth and productivity performance by elevating the nutritional value of wheat- and corn-based feeds [237]. Besides, xylanase supplementation has beneficial effects on egg quality, the egg productivity of laying hens, and their nutrient utilization during the consumption of wheat-based feeds [238]. Findings from recent studies raise the hypothesis that xylanase could play functional roles beyond increasing nutrient digestibility, but also enhancing intestinal health and positively modulating the intestinal microbiota of nursery pigs and broiler chickens (see review [239]).

The biotechnological potential of xylanases from thermophilic microbes and techniques for the optimization and production of these enzymes for various industrial applications are reviewed in ref. [240].

There are many scientific publications concerning the production of recombinant xylanases using the yeast *K. phaffii*. Let us examine some of those dealing with the production of thermostable enzymes. These are mainly enzymes from various fungal species, but there are also bacterial ones, either in a native form or in a mutant form with elevated thermal stability (see Table 3) [41,161,241,242,243,244,245,246,247,248,249,250,251,252,253].

Based on the data presented in Table 3, it can be concluded that to date, *K. phaffii* has been successfully used as a platform for the expression of heterologous genes to produce recombinant xylanases with high activity, stability at acidic pH, and high thermostability, e.g., xylanase enzyme preparations from the fungus *Bispora* sp. [247], *A. oryzae* [246], and the bacterium *T. maritima* [253]. Other examples of obtaining highly active xylanase preparations, mostly derived from fungi, are also available. 

For instance, the expression of the xylanase gene from the fungus *Paecilomyces thermophila* in *K. phaffii* has allowed researchers to obtain an enzyme preparation with an activity of 52,940 U/mL after culturing in a 5 L fermenter; the enzyme is stable up to 80 °C for 30 min [249].

Expression of the endo-1,4-β-xylanase gene from *Aspergillus oryzae* RIB40 in *K. phaffii* T07 has made it possible to obtain an enzyme preparation that hydrolyzes oat xylan with an activity of 258,240 U/mL after cultivation in a 5 L bioreactor [246]. Recombinant xylanase xAor has an optimal pH of 7.5, but the enzyme is highly active across a wide pH range (from 2.5 to 10) at temperatures of 35 to 85 °C with a maximum at 60 °C [246].

By means of genes of *M. thermophila*, two strains of *K. phaffii* have been created, which produce xylanases with a specific activity of 1533.7 and 1412.5 U/mg, and the levels of their activity after cultivation in a 7.5 L fermenter are 2010.4 and 2004.2 U/mL. Both xylanases show optimal activity at 60 °C and pH 6.0 and 7.0, and in terms of saccharification efficiency, are comparable to the commercial cellulase Celluclast 1.5 L [243].

There are examples of the production of mutant xylanases from bacteria *Bacillus pumilus* and *B. sonorensis* with elevated thermal stability; these enzymes, when *K. phaffii* serves as a host, have an activity approximately 40% higher relative to the enzyme produced by *E. coli* [254,255].

By means of thermostable xylanase Xyn11A from bacteria *Thermobifida fusca* as an example, a new strategy has been devised to increase the production of recombinant xylanase when *K. phaffii* is cultured in a 50 L bioreactor; this approach includes periodic feeding with glycerol and methanol, leading to the required cell density and desired xylanase activity. The resultant recombinant xylanase retains 82.5% of its activity after incubation at 80 °C for 50 min (pH 8.0) and possesses high stability across a wide range of temperatures (60–80 °C) [256].

The data presented above indicate the possible suitability of *K. phaffii* for the production of highly active thermostable xylanases, at a minimum by means of fungal or bacterial genes from *Aspergillus oryzae*, *A. niger*, *Bispora* sp., and *T. maritima*, which are appropriate sources of xylanase supplements for feed manufacture.

#### 4.3.2. Mannanases

Mannanase (mannan-endo-1,4-β-mannosidase, 1,4-β-D-mannanase) catalyzes random hydrolysis of β-1,4-mannosidic bonds in the backbone of β-mannans [257]. Mannans of various structures and compositions are renewable bioresources, widespread as constituents of lignocellulosic biomass from conifers and agricultural waste, as storage nonstarch polysaccharides in the endosperm and vacuoles of a wide variety of plants, and as the main component of the yeast cell wall. The major enzymes that cleave mannan are β-mannanases, β-mannosidases, and β-glucosidases. It is known that mannanases are synthesized in various bacteria (e.g., actinomycetes), fungi, plants, and animals. Microbial mannanases are mostly extracellular and are active in a wide range of pH and temperatures. In recent years, β-mannanases aroused interest due to a growing demand for stable and renewable bioresources and their industrial applications. β-Mannanases can be employed to produce manno-oligosaccharides as prebiotics via the enzymatic hydrolysis of plant cell walls and to eliminate antinutrients from corn- and soy-based feeds and are useful in the pulp/paper industry, detergents, and biofuels (see reviews: [258,259,260,261,262]).

Data on the positive influence of β-mannanase supplements on feed digestibility, pig productivity, and broiler growth are presented in refs. [263,264] and in a review [265]. Most of commercial β-mannanases are derived from bacteria and fungi owing to their high stability, cost-effectiveness, and ease of genetic manipulation [261].

The ability to assimilate mannan is present in the gram-positive bacterium *Lactobacillus casei* and some species from the genus *Bacillus* as well as in the gram-negative bacterium *Klebsiella oxytoca* [266,267,268]. Among fungi, this ability belongs to representatives of genera *Aspergillus*, *Streptomyces*, *Trichoderma*, and *Penicillium* and to *Lichtheimia ramose*, *Gloeophyllum trabeum*, *Coprinopsis cinerea*, and other microbes [269,270,271,272,273,274,275].

As for the manufacture of recombinant mannanases with the help of the yeast *K. phaffii*, there are quite a few such examples. Let us take a look at some of them with an emphasis on the thermal stability of the enzymes and resistance to acidic pH, which is important when enzymes are applied as a feed additive.

For instance, the gene of the highly thermostable β-mannanase from the bacterium *B. subtilis* has been successfully expressed in *K. phaffii*. During batch cultivation in a 50 L high-density fed-batch bioreactor, after 168 h induction with methanol, the highest activity of the recombinant β-mannanase was 5435 U/mL. The protein yield reached 3.29 mg/mL. The optimal pH and temperature of the purified enzyme proved to be 6.0 and 60 °C. The enzyme was found to be stable and active in a wide range of pH (2–8) and temperatures (20 to 100 °C), and after incubation at 100 °C for 10 min, it retained 58.6% of the maximal activity [276].

Nonetheless, most of the thermostable β-mannanases that are resistant to acidic pH have been isolated from various species of fungi, including those from the genus *Aspergillus*. The expression of genes of these β-mannanases under a strong promoter in the *K. phaffii* system, combined with a fermentation strategy involving high cell density, has enabled the production of recombinant enzymes with high activity, suitable for industrial use. Below are characteristics of some *K. phaffii* strains producing β-mannanase that are described in the literature. For instance, the overexpression of the β-mannanase gene from *Aspergillus kawachii* in *K. phaffii* during fermentation at high cell density allowed researchers to achieve a recombinant-enzyme activity of 11,600 U/mL, while the enzyme yield was 15.5 g/L; the maximal activity of the recombinant β-mannanase was registered at 80 °C and pH 2.0 [274].

There are also several examples of the successful expression of a β-mannosidase gene from various *A. niger* strains in *K. phaffii* [277,278,279,280,281]. All these recombinant mannanases have proven to be stable in a wide pH range and possess high thermal stability. For instance, at 80 °C, the mannanase from *A. niger* CBS 513.88 has a maximal activity of 3049 U/mL and is stable in the pH range from 1.5 to 11.0 [281]; mannanase from *A. niger* BK01 has a half-life of ~56 h at 70 °C and pH 4.0 [276]; and mannanase from *A. niger* LW-1 is stable in the pH range of 3.0–7.0 and has maximal activity at pH 3.5 and 70 °C [277].

There are known examples of the construction of mannanase producers from *K. phaffii* with the help of other fungal species, for example, *L. ramose*, *Neosartorya fischeri*, *Talaromyces leycettanus*, or others. For instance, β-mannosidase from the fungus *L. ramose* has maximal activity at pH 6 and 65 °C and is stable in the pH range of 3.0–8.5 (>80% of its activity after 10 min at 65 °C) [273]; β-mannanase from the thermophilic fungus *N. fischeri* P1 not only is resistant to elevated temperatures (up to 60 °C) and nonphysiological pH (exhibiting >20% of its activity at pH 2.0 and 9.0) but also has strong tolerance of sodium dodecyl sulfate (SDS) and proteases [160]; and β-mannanases from *Talaromyces leycettanus* have optimal activity at 85–90 °C and are very stable across a wide pH range of 3.0–10.0 [282].

#### 4.3.3. Cellulases and Laccases

Another family of enzymes has found good demand in agriculture for increasing the nutritional value of plant-based feeds: these are cellulases (including endoglucanases, cellobiohydrolases, and β-glucosidases) and laccases. 

Cellulases, together with hemicellulases, constitute almost 20% of the global market of industrial enzymes and have been used for several decades in various sectors of the economy, including food, feed, brewing, textile, detergent, pharmaceutical, pulp/paper, and other industries (see reviews: [178,283,284,285,286,287]. 

Cellulose is composed of repeating units of β-1,4-linked β-D-glucopyranose, and the complete degradation of cellulose to glucose is a complicated process requiring the synergistic action of three key cellulases: endoglucanase, which randomly cleaves internal bonds in the β-glucan chain; cellobiohydrolase, which acts on the ends of the polymer and releases cellodextrins; and β-glucosidase, which converts these cellodextrins into glucose.

The supplementation with exogenous β-glucanase, including in combination with xylanase, in poultry diets based on barley and wheat reduces the viscosity of the substance being digested and improves nutrient digestibility, egg productivity, and feed efficiency in laying hens [288,289,290]. The beneficial effects of an enzyme additive containing β-glucanase and xylanase are not limited to wheat/barley-based diets but are also seen with corn/soybean-based diets [291]. Cellulose-degrading enzymes are synthesized by bacteria, archaea, filamentous fungi, and some yeasts, although only a few of their species are capable of producing large amounts of extracellular cellulases [292,293,294]. The best-studied cellulase producers are among species of bacteria *Clostridium* and *Cellulomonas* and of fungi *Thermomonospora*, *Trichoderma*, and *Aspergillus* [295,296,297,298]. One of them is *Trichoderma reesei*, which generates high titers of extracellular thermostable cellulases and is industrially significant [299,300].

Laccases are oxidases that participate in the catabolism of lignin: an aromatic biopolymer that is one of the three major components of the plant cell wall. Laccases are common among fungi and higher plants, are found in some bacteria, and are abundant in white rot fungi [298].

Laccases have found applications in textile, pulp/paper, and food industries. They are used in the construction of biosensors for the detection and removal of toxic pollutants, in the design of biofuel batteries and medical diagnostics, and as biological remediation agents because they are quite effective at cleansing soil from herbicides, pesticides, and some explosives (see reviews: [301,302,303,304]). Aside from improving the nutritional value of feeds, the use of laccase as a food additive for feeding broiler chickens substantially reduces the residual level of antibiotics in broiler droppings and alleviates the dysbiosis of the intestinal microbiota when antibiotics are overused [177].

Various types of lignocellulosic biomass have high potential as a source of raw materials for the production of xylo-oligosaccharides, which, as prebiotics, have exceptional properties for the prevention of systemic diseases. All of this makes laccases beneficial, including their possible application to the improvement of nutrient bioavailability in (and digestibility of) animal feeds, especially in diets of monogastric animals [176,304]. Supplementation with a whole-cell product containing fungal laccase on the surface of *E. coli* cells as a feed additive for broiler chickens has allowed researchers to reduce the residual concentration of sulfonamide antibiotics in broiler droppings by 58% and to alleviate the dysbiosis of the intestinal microbiota owing to overuse of antibiotics [177]. 

The heterologous production of recombinant endoglucanases, cellobiohydrolases, β-glucosidases, and laccases by the yeast *K. phaffii* is discussed in detail in another review [174]. Among recent research articles, we can cite examples of the successful expression (in the *K. phaffii* system) of genes of endoglucanases from *Colletotrichum graminicola* [305,306], *Fomitopsis pinicola* [307], and *Penicillium funiculosum* [308]; the synthesis of recombinant cellobiohydrolases from *A. niger* [309], *Lentinula edodes* [310], and *Phanerochaete chrysosporium* [308]; the production of β-glucosidases from *Coptotermes formosanus* [311] and *Chaetomella raphigera* [62]; and the synthesis of laccases from *Pleurotus ostreatus* [312], *C. cinerea* [313], *Laccaria bicolor* [314], *Streptomyces coelicolor* [315], *Trametes versicolor* [316], and other microbes.

A comparative analysis of the characteristics of the β-endoglucanase from B. subtilis, when produced by either *K. phaffii* or *E. coli* Rosetta cells, indicates that the enzymatic activity in a cell lysate of *E. coli* reaches 20,010 U/mL, whereas this activity in the *K. phaffii* culture supernatant is only 2008 U/mL; however, the *K. phaffii* β-endoglucanase has significantly higher stability and retains 40% of its activity at 80 °C, whereas the activity of the enzyme from *E. coli* sharply drops at temperatures > 45 °C and is undetectable at temperatures > 70 °C [317].

The characteristics of endoglucanase E1 from Acidothermus cellulolyticus and its production in different expression systems are reported to vary too. For instance, in *E. coli*, a substantial amount of the enzyme is produced, but it has low specific activity; when expressed in Streptomyces lividans, the enzyme accumulates in the culture at ~100 mg/L, but the medium contains a high concentration of other proteins, causing excessive frothing during fermentation and requiring additional purification. The production of endoglucanase E1 in the *K. phaffii* system has turned out to be the most successful, where the enzyme yield is 550 mg/L [318].

A comparative analysis of characteristics of the endoglucanase II and cellobiohydrolase II from Trichoderma reesei, when produced by either *K. phaffii* or Yarrowia lipolytica, indicates that the specific activity enzymes from Y. lipolytica were approximately 1.5 times greater than those of enzymes produced by *K. phaffi*; however, the maximum level of their production in *Y. lipolytica* was almost an order of magnitude lower than that in *K. phaffi* [319]. These data suggest that the preferred host for cellulase production is *K. phaffii*.

### 4.4. K. phaffii as a Protease Producer

Another class of enzymes is needed in agriculture to increase the nutritional value of plant feed: these are proteases, which can hydrolyze the peptide bonds of proteins, including plant proteins, via various catalytic mechanisms. Protease supplementation has been shown to positively affect the digestibility of grain-based feeds and the productivity of laying hens [320,321] as well as chick growth and the nutritional value of soybean meal [322]. This class of enzymes is one of the three largest among industrial enzymes whose global market is growing every year. Of the 60% of enzymes sold worldwide, proteases represent 20% (cited by [323]). The molecular and biotechnological issues of the practical application of these enzymes in manufacturing are presented in some reviews [323,324].

It was stated above that thermostable and acidophilic enzymes are desirable as feed additives. The source of acid proteases is mainly various species of filamentous fungi Aspergillus, and some of these enzymes are not only resistant to high temperatures [325,326,327] but also have exceptionally high specific activity [328].

Regarding enzymes that hydrolyze plant proteins, *K. phaffii* has also been successfully utilized to create strains that synthesize thermostable proteases that will be helpful in animal husbandry for feed manufacture and the improvement of feed quality. An example is the *K. phaffii* strains secreting recombinant proteases from *A. niger* [326,327,328], *Bispora* sp. MEY-1 [329], *Aspergillus pseudotamarii* [330], or *Tritirachium album* [331].

For instance, aspartic protease from A. niger (aspergillopepsin A-like endopeptidase) has proven to be exceptionally active (specific activity 40,000 ± 1800 U/mg), has a pH and temperature optima of 3.5 and 60 °C, is stable for 60 min at 50 °C, and hydrolyzes the following commercial substrates with high efficiency (in descending order): hemoglobin > defatted soy flour > gluten > gelatin > skim milk powder [328].

The recombinant protease pAsP that has been prepared through the optimization of the gene sequence of the NpI enzyme from A. pseudotamarii possesses exceptionally high specific activity toward casein (7,657,000 U/mg), quite efficiently hydrolyzes azocasein (specific activity 25,344 U/mg), and is less efficient at hydrolyzing hemoglobin (specific activity 2320 U/mg). pAsP has the highest activity between 50 and 60 °C [330]. 

## 5. Conclusions

The data presented above indicate that *K. phaffii* has been extensively used as an efficient platform for heterologous recombinant protein production due to its GRAS status, rapid growth rate on various inexpensive substrates, including, in addition to methanol, glycerin, sorbitol, glucose, mannitol, sucrose, xylose, and cellobiose, as well as the ability for high-cell-density fermentation [27,29]. 

To date, practical approaches have been developed and technological solutions have been described for the efficient expression of recombinant proteins in *K. phaffii* [31,32]. 

**Figure 4:**
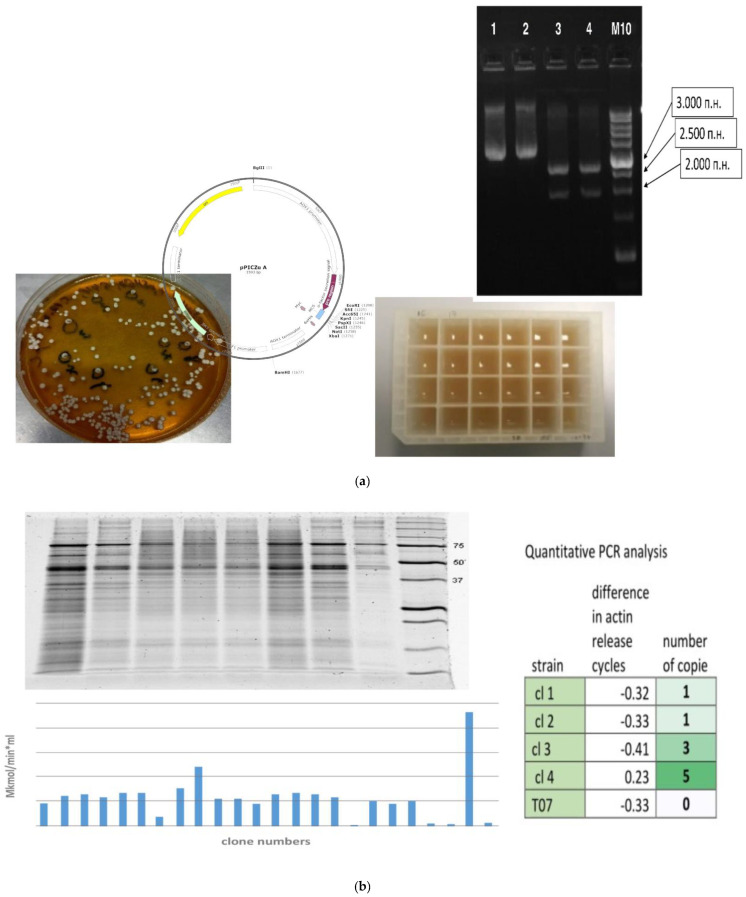

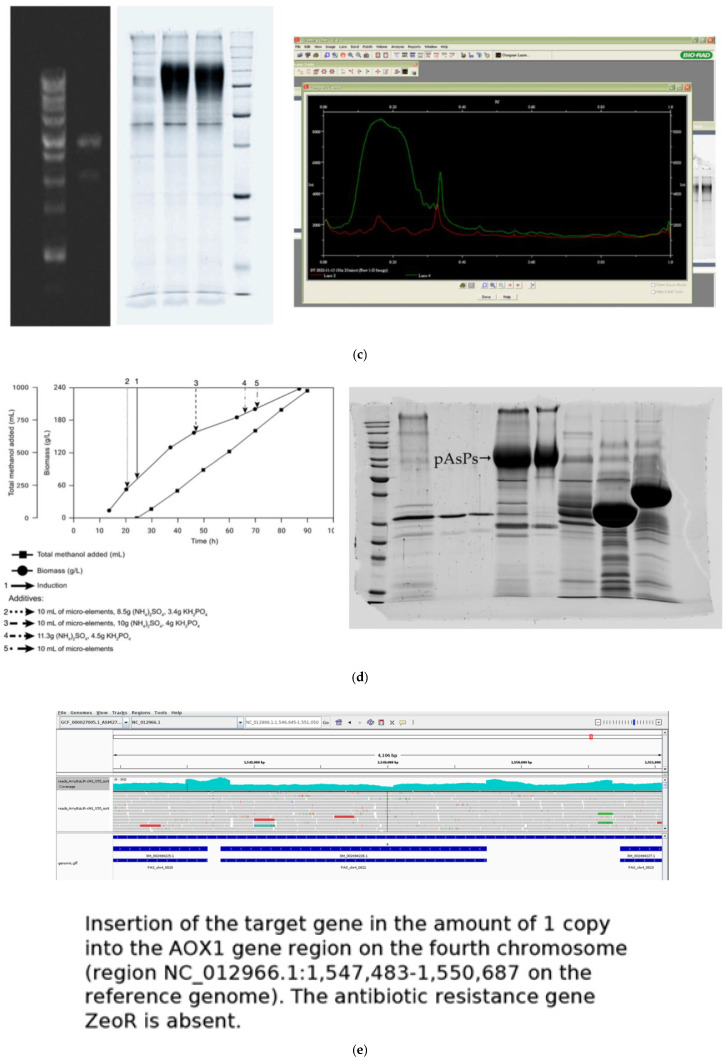

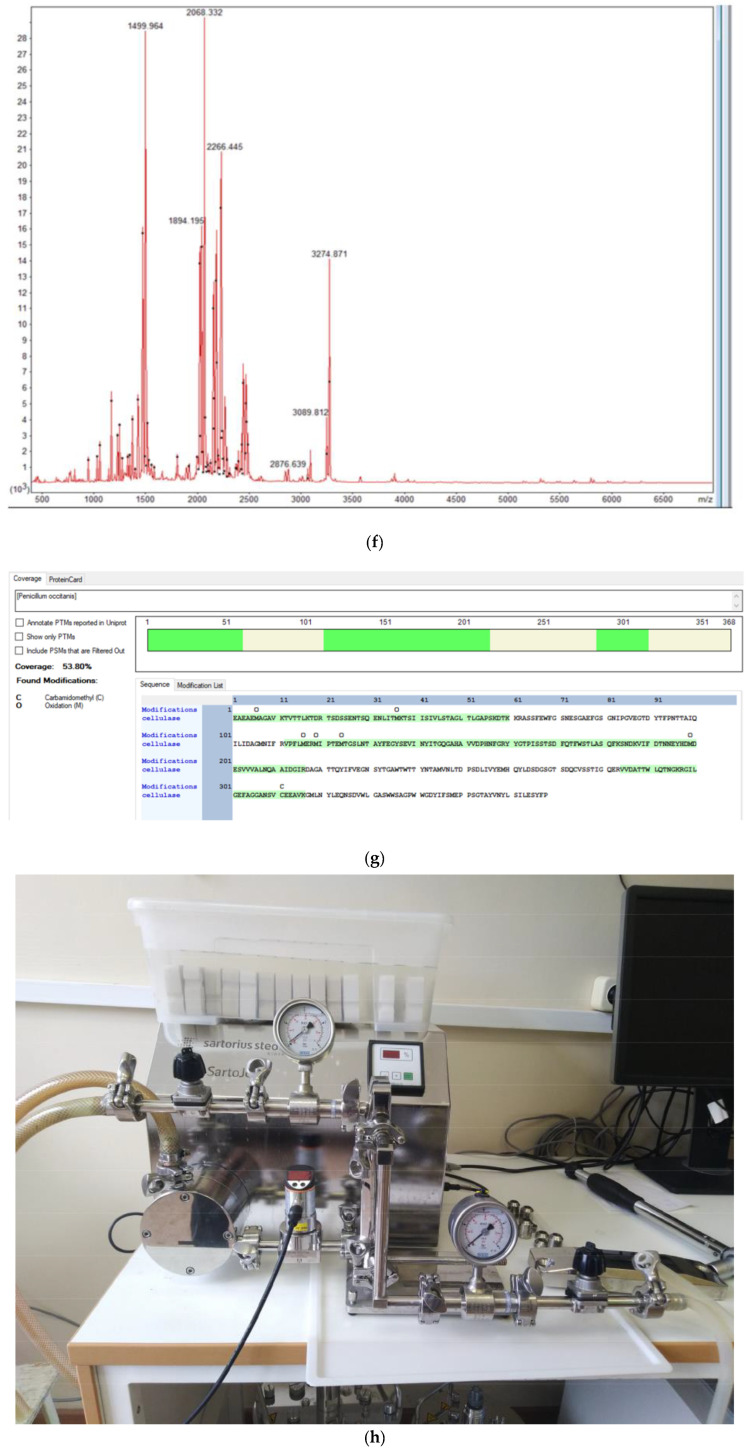

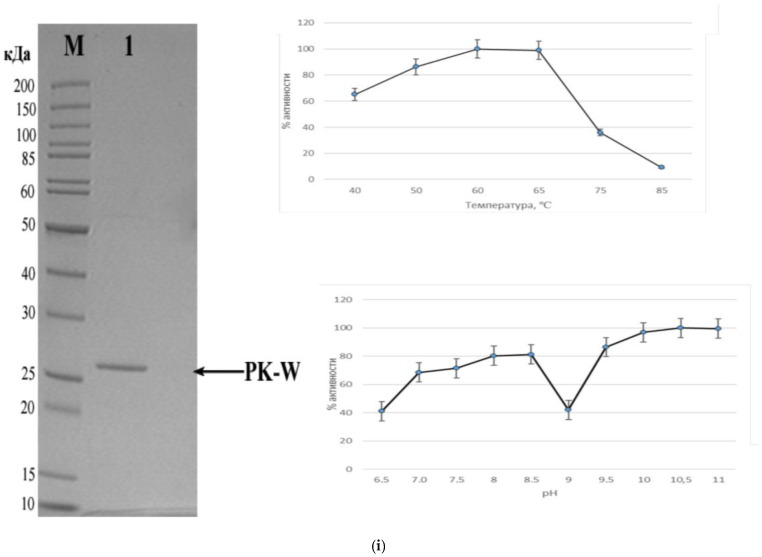
The common scheme of producing recombinant strains of enzyme producers based on the *K. phaffii* genome. (**a**) Target gene cloning; (**b**) analysis of recombinant clones for the presence of protein and enzyme activity; (**c**) electrophoretic analysis of the mannanase preparation from the culture liquid of recombinant clone; (**d**) analysis accumulation of biomass and protein during cultivation of selected clones in a bioreactor; (**e**) full genomic sequencing of the nucleotide sequence of the genome of the strain producing the target protein; (**f**) analysis of the culture fluid of the producer line (general view of the MS1 mass spectrogram containing the peptide mixture obtained from the culture liquid); (**g**) the fragment peptide sequence founding in culture fluid samples of the recombinant strain *K. phaffii* T07 identifying the structure with the specified putative amino acid sequence with a convergence of 58.22%; (**h**) protein and enzyme preparations by tangential diffusion and liquid chromatography methods; (**i**) characteristics of purified and lyophilized proteinase K, dependence of enzyme activity on pH and temperature. Adapted from [246,330,331,332].

Figure 4 shows the main stages of the process of obtaining recombinant enzyme-producing strains based on the *K. phaffii* genome and the experimental approaches used in cloning the target gene, including cloning the target gene into the genome; selection by assessing the obtained clones based on the activity of the target protein, the results of quantitative PCR analysis of its genes in the genome and the accumulation of its fraction in cultural fluid; semi-quantitative analysis of its content in the culture liquid by electrophoresis of the target protein; analysis accumulation of the biomass and protein during the cultivation of selected clones in a bioreactor; full genomic sequencing of the nucleotide sequence of the genome of the strain producing the target protein; identification of the amino acid sequence of the target protein in the process of expression and secretion of this gene by the yeast genome when cultivating the producer strain in a bioreactor; obtaining protein and enzyme preparations by tangential diffusion and liquid chromatography methods; obtaining basic biochemical characteristics of pretarates such as dependence of enzyme activity on pH and temperature; and obtaining highly purified drugs using high-pressure liquid chromatography methods [246,330,331,332].

The cited studies also point to the high potential of *K. phaffii* for industrial applications in terms of its secretion capacity and some advantages from the standpoint of protein folding and glycosylation, which positively affect enzymes’ properties and thermal stability. Thus, several of the enzymes analyzed above, produced using *K. phaffii*, (i) exhibit higher thermostability compared to when other expression systems are used, as demonstrated for phytase AppA *E. coli* [198], glucoamylase from *Aspergillus awamori* [235], β-endoglucanase from B. subtilis [317]; and (ii) exhibit higher production levels, as demonstrated for α-amylase from *Alkalimonas amylolytica* [283], endoglucanase E1 from Acidothermus cellulolyticus [218], and endoglucanase II and cellobiohydrolase II from Trichoderma reesei [319].

A series of enzyme preparations with high activity and unique stability at high temperatures and low pH were obtained using *K. phaffii*, which is important for their application in pelleted animal feed. These are phytase *E. coli* [198], and xylanases from the fungus *Bispora* sp. [247] and *T. maritima* [253].

So the data outlined above are evidence of the successful use of *K. phaffii* for the heterologous production of almost all the major enzymes important for increasing the nutritional value of feed, including the most popular ones: thermostable phytases [198], glucoamylases [229,230,231,232,233,234], and α-amylases [223,224,225], including the co-expression of glucoamylase and α-amylase genes [230] as well as for the heterologous synthesis of xylanase [246,247,253], mannanase [276,277,279,281,282,283,285,332], laccase [314], cellulases [316], and proteases [326,327,328,329,330,331].

Currently, there is no doubt about the need for the use of enzyme preparations, including phytase, amylase, cellulase, xylanase, mannanase, protease, and others, to improve feed digestibility and increase its nutritional value [178,182,183,184,320,321], especially for birds and animals with a single-chambered stomach [179,180,181,185,186,187,237,238,263,264,265,320,321].

Data also accumulate about the potential positive impact of enzyme additives on animal health [180,181,185,186,187], problems with which arise, including as a result of low feed digestibility [181].

The global animal feed enzyme market is expected to grow at a compound annual growth rate (CAGR) of 5.0% during the period of 2022–2028 (cited in [178]). These data indicate that enzymes are becoming an important ingredient in feed production.

Over the past 20 years, the use of enzymes in various industrial sectors has been steadily increasing. Currently, no production process, from glucose syrups and brewing to wastewater treatment, can operate without the use of enzymes.

Therefore, the issues of obtaining inexpensive and effective enzyme preparations that are safe for both human and animal health remain relevant and require attention from the scientific community to develop high-productivity-producing strains and new sources of enzyme preparations and proteins.

## Figures and Tables

**Table 1:** The functions and examples of co-overexpression of certain proteins involved in the protein secretion process and UPR.

Protein Name	Description	Effects of Protein Co-Overexpression on Target Protein Production in *K. phaffii* *
Bfr2	Essential protein possibly involved in secretion; multicopy suppressor o1f sensitivity to brefeldin A [136]	The secretion of Fab antibody fragment showed a 1.4-fold increase [136]
Bmh2	14-3-3 protein isoform; binds proteins and DNA, involved in regulation of many processes, including exocytosis and vesicle transport, protein exit from the ER [136,137]	The secretion of Fab antibody fragment showed a 1.4-fold increase [136]
Cup5	Vacuolar ATP synthase proteolipid subunit (EC 3.6.3.14) required for vacuolar acidification. Important for copper and iron metal ion homeostasis [131]	The secretion of Fab antibody fragment showed a 1.7-fold increase [136]
Ero1	Pdi oxidase, protein-thiol disulfide exchange; required for oxidative protein folding in the ER [131,136]	The secretion of Fab antibody fragment showed a 1.4-fold increase [136]. The secretion of human albumin (HSA) fusion protein IL2-HSAa showed a 2.3-fold increase [23]. Phytase production was not improved by sole co-overexpression of Ero1, while the co-overexpression Pdi1 and Ero1 improved phytase production by 1.21 ± 0.06-fold [56]
Hac1	bZIP transcription factor that regulates the unfolded protein response via UPRE binding and membrane biogenesis [136]	Increase, decrease, or have no effect on the production of the target protein [136]
Kar2	Binding protein BiP, ATPase involved in protein import into the ER, acts as a chaperone to mediate protein folding in the ER; regulates the unfolded protein response [131]	The secretion of Fab antibody fragment showed a 1.5-fold increase [133]. The secretion of human albumin (HSA) fusion protein IL2-HSAa showed a 1.9-fold increase [23] No effect on the phytase AppA production [56]
Kin2	Serine/threonine protein kinase involved in regulation of exocytosis; localizes to the cytoplasmic face of the plasma membrane [131]	The secretion of Fab antibody fragment showed a 1.5-fold increase [136]
Pdi1	Protein disulfide isomerase, multifunctional protein resident in the ER lumen, essential for the formation of disulfide bonds in secretory and cell-surface proteins [131]	Increases the production of heterologous proteins when *K. phaffii* serves as a cell host [23,136,137,138]
Sbh1	A subunit of the Sec61p ER translocation complex [139]	No effect on the IgG production [139]
Sec1	Interacts with vesicle trafficking between the Golgi and cell membrane [56]	The secretion of human albumin (HSA) fusion protein IL2-HSAa 2.5-fold increase [23]. No effect on phytase AppA production [56]
Sly1	Regulates ER-Golgi trafficking and Sec1 interacts with vesicle trafficking between the Golgi and cell membrane [56]	The secretion of human albumin (HSA) fusion protein IL2-HSAa showed a 1.9-fold increase [23]. No effect on the phytase AppA production [56]
Ssa4	Cytoplasmic member of the HSP70 family; highly induced upon stress; plays a role in SRP-dependent cotranslational protein-membrane targeting and translocation [131]	The secretion of human albumin (HSA) fusion protein IL2-HSAa showed a 1.9-fold increase [23]. No effect on the phytase AppA production [56]
Sse1	ATPase that is a component of the Hsp90 chaperone complex; binds unfolded proteins; member of the HSP70 family; localized to the cytoplasm [131]	The secretion of Fab antibody fragment showed a 1.4-fold increase [136]

* The third column provides examples of co-overexpression of proteins and its influence on the target protein output. However, these data cannot be extrapolated to other experiments. Moreover, with a larger number of experiments involving a specific protein (for example, HAC1 protein), we observe that the results are unpredictable and seemingly depend on each individual case. Moreover, it is worth noting that the data on this issue are quite fragmented and too scarce to create a comprehensive picture. Additionally, there is no widely accepted practice of publishing negative results, which also hinders the systematization of data.

**Table 2:** Characteristics of phytase preparations produced by *K. phaffii*.

Gene Origin	Specific Activity, U/mg	Temperature Optima, °C	pH Optima, (Range)	Thermostability	Productivity, mg/mL	Reference
*Cronobacter turicensis*	1705	50	4.5 (2–8)	65%, 5 min, 60 °C	–	[36]
*Aspergillus niger*	142	60	2.5 and 5.5	*t*_1/2_ 10 min, 80 °C	6.1	[40]
*E. coli*	19,880	–	5.5	30%, 99 °C, 60 min	0.04	[198]
*Shigella* sp. CD2	967	60	5.5 (3.5–6.5)	>60% 30 min, 70 °C	–	[203]
*Citrobacter braakii*	3.5 × 10^6^	55	4.5 (2–7)	–	3.2	[205]
*C. amalonaticus*	3548	50	4.5	–	4.2	[206]
*C.* *gillenii*	1577	55	4.5 (3–6)	50%, 65 °C, 5 min	–	[207].
*Yersinia intermedia*	3960	55	4.5 (2–6)	>50%, 80 °C, 15 min	–	[208]

**Table 3:** Characteristics of xylanase preparations produced by *K. phaffii*.

Gene Origin	Activity	Temperature Optima, °C	pH Optima	Stability	Reference
*Chaetomium* sp. CQ31	10,017 U/mL 1208 U/mg	85	6.5 (5.0–9.5)	stable up to 60 °C	[241]
*Gloeophyllum trabeum*	1205 ± 28 U/mg	75	4.5	>60%, 70 °C, 30 min	[242]
*Myceliophthora* *thermophila*	2010 U/mL	60	6.0	60%, 70 °C, 30 min 70%, pH 2–12, 60 min	[243]
*Aspergillus sulphureus*	1684 U/mL 218 U/mg	55	3.0 (2–4)	t1/2 39.6 min, 60 °C t1/2 9.5 min, 70 °C	[244]
*Aspergillus sulphureus*	180 U/mL	70	5.0 (4.5–6.5)	50% 30 min, 60 °C	[245]
*Aspergillus oryzae*	258,240 U/mL	60	7.5 (2.5–10)	stable up to 85 °C	[246]
*Bispora* sp. MEY–1	73,400 U/mL	85	3 and 4.5–5.0	100%, 80 °C, 60 min >87%, 90 °C, 10 min t1/2 45 h, 80 °C t1/2 3 h, 85 °C 80%, pH 1.5–6.0, 1 h	[247]
*Trichoderma asperellum*	393 ± 18 U/mg	50	3 (2–5)	>60% 30 min, 55 °C	[248]
*Paecilomyces thermophila*	52,940 U/mL 6536 U/mg	75	7 (4.5–10)	stable up to 80 °C	[249]
*Aspergillus niger*	52,940 U/mg	50	5.0 (2.2–7.0)	>40%, 90 °C, 10 min	[250]
*Thermobifida fusca*	515.8 U/mg	80	5.0–9.0	>60%, pH 6–9, 60 min >60%, 80 °C, 30 min	[251]
*Streptomyces* sp. FA1	3925 U/mL 289 U/mg	55	5.0	–	[252]
*Thermotoga maritima*	40,020 U/mL 3962 U/mg	100	5.5	90%, pH 4–11, 30 min >90%, 90 °C, 30 min	[253]

## Data Availability

The authors confirm that the data supporting the findings of this study are available within the article.
